# Guanosine regulates adenosine levels in the kidney

**DOI:** 10.14814/phy2.12028

**Published:** 2014-05-28

**Authors:** Edwin K. Jackson, Dongmei Cheng, Zaichuan Mi, Delbert G. Gillespie

**Affiliations:** 1 Department of Pharmacology and Chemical Biology, School of Medicine, University of Pittsburgh, Pittsburgh, Pennsylvania

**Keywords:** 8‐Aminoguanine, adenosine, guanosine, inosine, kidney, purine nucleoside phosphorylase

## Abstract

In cell culture, extracellular guanosine increases extracellular adenosine by attenuating the disposition of extracellular adenosine (*American Journal of Physiology* – *Cell Physiology* 304: C406–C421, 2013). The goal of this investigation was to determine whether this “guanosine–adenosine mechanism” is operative in an intact organ. Twenty‐seven isolated, perfused mouse kidneys were subjected to metabolic poisons (iodoacetate plus 2,4‐dinitrophenol) to cause energy depletion and thereby stimulate renal adenosine production. Adenosine levels in the renal venous perfusate increased from a baseline of 36 ± 8 to 499 ± 96, 258 ± 50, and 71 ± 13 nmol/L at 15, 30, and 60 min, respectively, after administering metabolic poisons (% of basal; 1366 ± 229, 715 ± 128, and 206 ± 33, respectively). Changes in renal venous levels of guanosine closely mirrored the time course of changes in adenosine: baseline of 15 ± 2 to 157 ± 13, 121 ± 8, and 50 ± 5 nmol/L at 15, 30, and 60 min, respectively (% of basal; 1132 ± 104, 871 ± 59, and 400 ± 51, respectively). Freeze‐clamp experiments in 12 kidneys confirmed that metabolic poisons increased kidney tissue levels of adenosine and guanosine. In eight additional kidneys, we examined the ability of guanosine to reduce the renal clearance of exogenous adenosine; and these experiments revealed that guanosine significantly decreased the renal extraction of adenosine. Because guanosine is metabolized by purine nucleoside phosphorylase (PNPase), in another set of 16 kidneys we examined the effects of 8‐aminoguanine (PNPase inhibitor) on renal venous levels of adenosine and inosine (adenosine metabolite). Kidneys treated with 8‐aminoguanine showed a more robust increase in both adenosine and inosine in response to metabolic poisons. We conclude that in the intact kidney, guanosine regulates adenosine levels.

## Abstract (precis)

In cell culture, extracellular guanosine increases extracellular adenosine by attenuating the disposition of extracellular adenosine (*American Journal of Physiology* – *Cell Physiology* 304: C406–C421, 2013). The goal of this study was to determine whether the “guanosine–adenosine mechanism” is operative in an intact organ. In isolated, perfused mouse kidneys, inhibition of energy production induced changes in renal venous levels of guanosine that closely mirrored the time course of changes in adenosine, and freeze‐clamp experiments confirmed that metabolic poisons similarly increased kidney tissue levels of adenosine and guanosine. Moreover, exogenous guanosine significantly decreased the renal extraction of exogenous adenosine, and inhibition of purine nucleoside phosphorylase (metabolizes guanosine) augmented the effects of energy depletion on renal levels of both guanosine and adenosine. We conclude that in the intact kidney, guanosine regulates adenosine levels.

## Introduction

Extracellular adenosine modulates cellular function by activating G‐protein‐coupled cell surface receptors, namely A_1_, A_2A_, A_2B_, and A_3_ receptors (Grenz et al. 2011). Therefore, it is important to understand the determinants of extracellular adenosine levels. Our recent studies (Jackson and Gillespie 2013; Jackson et al. 2013) in cell culture systems (preglomerular vascular smooth muscle cells, glomerular mesangial cells, cardiac fibroblasts, kidney epithelial cells, aortic and coronary artery vascular smooth muscle cells, and coronary artery endothelial cells) show that (1) extracellular guanosine inhibits the disposition of adenosine from the extracellular compartment; (2) metabolic poisons to inhibit glycolysis (iodoacetate) plus oxidative phosphorylation (2,4‐dinitrophenol) increase extracellular levels of both endogenous adenosine and guanosine; (3) inhibition of purine nucleoside phosphorylase (PNPase; converts guanosine to guanine; Giblett 1985; Seegmiller 1985) augments the effects of metabolic poisons on extracellular levels of both guanosine and adenosine; (4) the effects of guanosine on extracellular adenosine levels are not mimicked nor attenuated by inhibition of the major systems that are known to metabolize (i.e., adenosine kinase, adenosine deaminase, *S*‐adenosylhomocysteine hydrolase) or transport (equilibrative nucleoside transporters or concentrative nucleoside transporters) adenosine; and (5) extracellular guanosine augments the ability of extracellular adenosine to regulate cell proliferation via adenosine receptors. Therefore, the evidence thus far suggests that extracellular guanosine regulates extracellular adenosine levels (called the “guanosine–adenosine mechanism”) thus allowing for an indirect signaling role for guanosine. By “indirect” we mean that guanosine signals by increasing extracellular adenosine which in turn acts on specific cell surface G‐protein‐coupled adenosine receptors (which are well‐known to exist), rather than by direct signaling via specific cell surface guanosine receptors (which may [Traversa et al. 2003] or may not [Thauerer et al. 2012] exist). An important unanswered question, however, is whether this “guanosine–adenosine mechanism” is an artifact of cell culture model systems or actually exists in intact organs. The present study addresses this question by examining the guanosine–adenosine mechanism hypothesis in an intact organ, namely the isolated, perfused mouse kidney.

## Methods

### Chemicals

All chemical were from Sigma–Aldrich (St. Louis, MO).

### Animals

Male C57BL/6 mice (10–12 weeks of age; Taconic Farms, Germantown, NY) were housed at the University of Pittsburgh Animal Facility and provided Pro Lab RHM 3000 rodent diet (PMI Feeds, Inc., St. Louis, MO). All procedures were approved by the University of Pittsburgh's IACUC. The experiments conformed to the *Guide for the Care and Use of Laboratory Animals* (NIH Publication No. 85‐23, revised 1996).

### Isolated, perfused mouse kidney

After anesthesia with Inactin (100 mg/kg, i.p.), the bladder was cannulated (PE‐50) and the right ureter was ligated, thus permitting urine to exit the left kidney. Cannulas (PE‐50 and PE‐10, respectively) were inserted into the distal vena cava and aorta, with the tip of the cannulas positioned near the origins of the left renal vein and artery. During the isolation procedure, renal perfusion was maintained by pumping Tyrode's solution through the left renal artery. Branching vessels of the aorta and vena cava that were near the renal vein and left renal artery were tied, and the vena cava and aorta were ligated. The left kidney was rapidly secured in a kidney perfusion system (Hugo Sachs Elektronik‐Harvard Apparatus GmbH; March‐Hugstetten, Germany) and was perfused (single pass mode) at 1.5 mL/min (normal mouse renal blood flow; Oppermann et al. 2007) with Tyrode's solution of the following composition: NaCl, 137 mmol/L; KCl, 2.7 mmol/L; CaCl_2_, 1.8 mmol/L; MgCl_2_, 1.1 mmol/L; NaHCO_3_, 12 mmol/L; NaH_2_PO_4_, 0.42 mmol/L; d(+)‐glucose, 5.6 mmol/L; pH, 7.4; osmolality, 295 mOsm/kg. Before entering the kidney, the Tyrode's solution was gassed with 95% O_2_/5% CO_2_, was warmed to a temperature of 37°C, and was propelled via a roller pump through an oxygenator (95% oxygen/5% carbon dioxide), particle filter, Windkessel, heat exchanger, and bubble remover. An in‐line Statham pressure transducer (model P23ID; Statham Division, Gould Inc., Oxnard, CA) was used to measure perfusion pressure, which was recorded on a Grass polygraph (model 79D; Grass Instruments, Quincy, MA).

### Sample collection and processing

In some experiments, perfusate exiting the renal vein was collected, immediately placed in boiling water for 90 sec to denature any enzymes in the perfusate and then frozen at −80°C for later analysis of purines by ultraperformance liquid chromatography–tandem mass spectrometry (LC‐MS/MS) as described below. Given that the average weight of our mouse kidneys was 0.18 g, and assuming that 33.3% of tissue volume was extracellular, 25% of the extracellular volume was intravascular, the time required for the intravascular compartment to be replaced with fresh perfusate was approximately 0.6 sec. Therefore, monitoring renal venous levels allowed us to monitor intravascular changes nearly in real time.

In other experiments, while the isolated, perfused kidney was perfusing, the whole kidney was dropped into liquid nitrogen and compressed with a metal clamp that was kept in liquid nitrogen until use. Then the kidney was placed in 5 mL of 1‐propanol (−20°C) and rapidly cut into small pieces, and the tissue and 1‐propanol were placed in a 10‐mL test tube and the sample was homogenized. One milliliter of the 1‐propanol/tissue mixture was centrifuged, and the supernatant was collected, taken to dryness with a sample concentrator and reconstituted in 0.2 mL of water. Next the sample was filtered to 30 kDa using a Microcon YM‐30 centrifugal filter unit (Millipore; Billerica, MA) and then frozen at −80°C for later analysis of purines by LC‐MS/MS as described below.

### Analysis of purines

The LC‐MS/MS analytical system consisted of an Accela ultraperformance liquid chromatograph (ThermoFisher Scientific, San Jose, CA) interfaced with a TSQ Quantum‐Ultra triple‐quadrupole mass spectrometer (ThermoFisher Scientific). The column was an Agilent Zorbax eclipse XDB‐C‐18 column (3.5 *μ*m beads; 2.1 × 100 mm) and samples were introduced into the mass spectrometer using a heated electrospray ionization source. The LC‐MS/MS system operated in the selected reaction monitoring mode. The mobile phase (pumped at 300 *μ*L/min) was a gradient of two buffers (Buffer A: 0.1% formic acid in water; Buffer B: 0.1% formic acid in methanol). The gradient (A/B) was 0–2 min, 98.5%/1.5%; 2–4 min, 98%/2%; 5–6 min, 92%/8%; 7–8 min, 85%/15%; 9–11.5 min, 98.5%/1.5%. Four transitions were monitored: for adenosine, 268→136; for inosine, 269→137; for guanosine, 284→152; and for ^13^C_10_‐adenosine (internal standard), 278→141.

### Statistical analysis

Statistical analysis was performed with 1‐factor or 2‐factor analysis of variance (ANOVA) with post hoc comparisons using a Fisher's Least Significant Difference (LSD) test if main‐effect or interaction‐effect *P*‐values justified post hoc tests. Comparisons between two groups were performed with unpaired or paired Student's *t*‐tests as appropriate. The criterion of significance was *P* < 0.05. All values in text and figures are means and SEMs.

## Results

To determine the relationship between adenosine, inosine (adenosine metabolite), and guanosine levels in the mouse kidney, mouse kidneys (*n* = 27) were isolated and perfused with Tyrode's solution, allowed a 1‐h rest period, and then treated with metabolic poisons to block energy production and stimulate adenosine synthesis. In this regard, we employed iodoacetate (50 *μ*mol/L) to block glycolysis (McKee et al. 1965, 1968; Konings 1971) plus 2,4‐dinitrophenol (50 *μ*mol/L; Joel et al. 1967; Kaminsky and Kosenko 1987; Desquiret et al. 2006) to inhibit oxidative phosphorylation. We used this approach rather than removing oxygen from the perfusate or discontinuing perfusion because the former method gives rise to variability depending on the time required for oxygen depletion from the Tyrode's solution and the latter approach does not allow for collection of renal venous perfusate during energy depletion. In contrast, giving metabolic poisons permits rapid, reliable, and reproducible energy depletion while maintaining perfusion constant.

**Figure 1.**
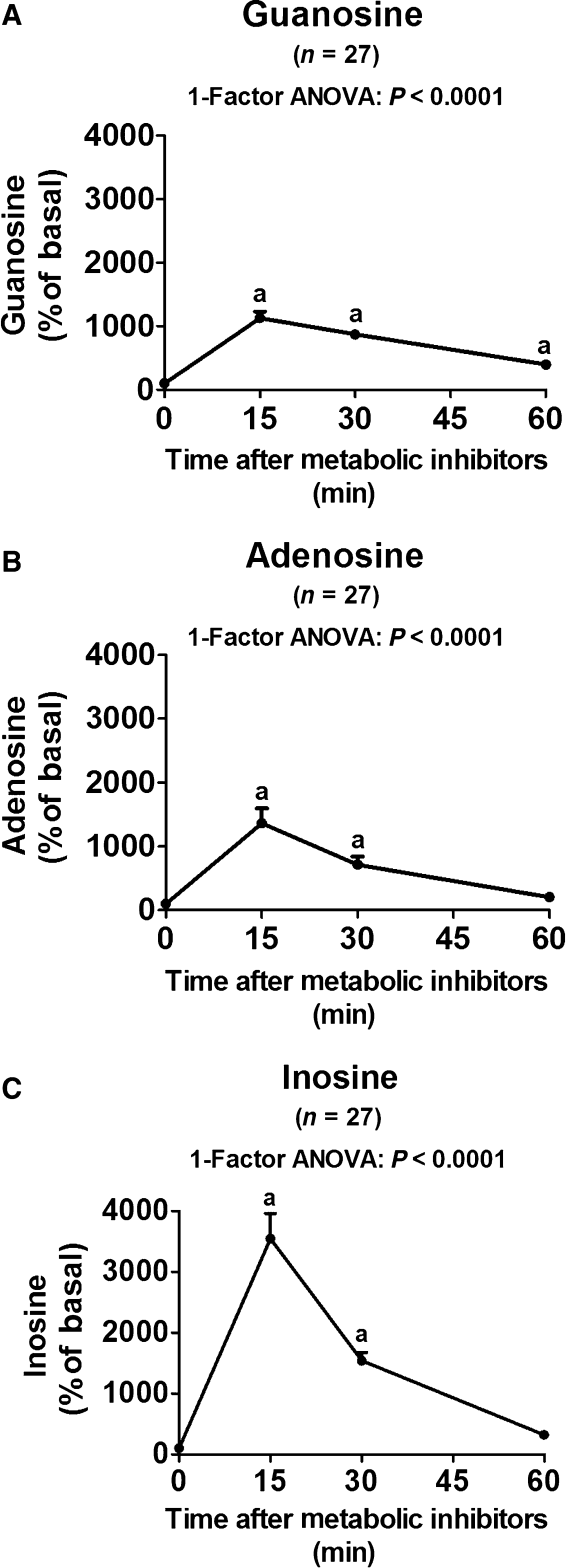
Line graphs summarize the time‐dependent effects of metabolic inhibitors (iodoacetate and 2,4‐dinitrophenol, each at 50 *μ*mol/L) on changes (% of basal) in concentrations of (A) guanosine, (B) adenosine, and (C) inosine in the renal venous perfusate in isolated, perfused mouse kidneys. Basal values were 15 ± 2, 36 ± 8, and 86 ± 9 nmol/L for guanosine, adenosine, and inosine, respectively. Values represent means and SEMs, and “a” indicates significantly different from the time 0 levels.

Just before administering the metabolic poisons, perfusion pressure was 40 ± 1 mmHg, and increased only slightly by 15 min to 45 ± 2 mmHg (*P* < 0.05). Because severe renal hypoxia is known to increase both preglomerular and postglomerular resistances (Denton et al. 2002), the slight increase in renal perfusion pressure most likely reflects a vasoconstrictive response of both vascular compartments. However, at 30 and 60 min, perfusion pressure was no longer elevated (40 ± 2 and 39 ± 1 mmHg, respectively). Basal renal venous levels of guanosine, adenosine, and inosine (adenosine metabolite) were 15 ± 2, 36 ± 8, and 86 ± 9 nmol/L, respectively. At 15, 30, and 60 min following administration of metabolic poisons, the concentrations of guanosine, adenosine, and inosine in the renal venous perfusate increased (Fig. 1). Expressed as % of basal (time zero) concentrations, guanosine increased to 1132 ± 104, 871 ± 59, and 400 ± 51; adenosine increased to 1366 ± 229, 715 ± 128, and 206 ± 33; and inosine increased to 3545 ± 413, 1542 ± 134, and 323 ± 41 (all values % of basal at 15, 30, and 60 min, respectively). These results demonstrate the efficacy of using metabolic poisons to stimulate the production of guanosine, adenosine, and inosine in the perfused mouse kidney and show that the time course of increase in all three purines is similar.

**Figure 2.**
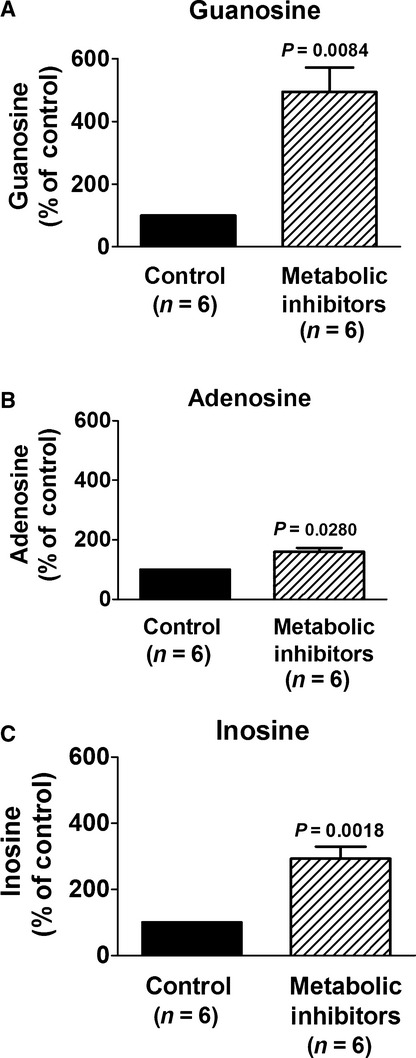
Bar graphs show the effects of metabolic inhibitors (iodoacetate and 2,4‐dinitrophenol, each at 50 *μ*mol/L for approximately 15 min) on changes (% of control kidneys) in kidney tissue concentrations of (A) guanosine, (B) adenosine, and (C) inosine in isolated, perfused mouse kidneys. In the absence of metabolic poisons, kidney levels of guanosine, adenosine, and inosine were 0.26 ± 0.06, 2.02 ± 0.40, and 0.69 ± 0.19 nmol/mg, respectively. Values represent means and SEMs, and *P*‐values are for unpaired Student's *t*‐tests comparing control kidneys to kidneys treated with metabolic inhibitors.

To determine the relationship between guanosine, adenosine, and inosine in the local tissue environment in the kidney, in a separate set of experiments we examined the effects of energy depletion on tissue levels of purines in isolated, perfused mouse kidneys. In these experiments, metabolic poisons were administered into the perfusate for approximately 15 min. Then the kidneys were dropped, while still perfusing, directly into liquid nitrogen and processed as carefully as possible to preserve a “snapshot” of tissue levels of guanosine, adenosine, and inosine at the instant of freezing. Six kidneys did not receive metabolic poisons in the perfusate (controls) and six kidneys were treated with iodoacetate plus 2,4‐dinitrophenol. In the absence of metabolic poisons, kidney levels of guanosine, adenosine, and inosine were 0.26 ± 0.06, 2.02 ± 0.40, and 0.69 ± 0.19 nmol/mg, respectively. Metabolic poisons significantly increased tissue levels of guanosine, adenosine, and inosine (Fig. 2).

**Figure 3.**
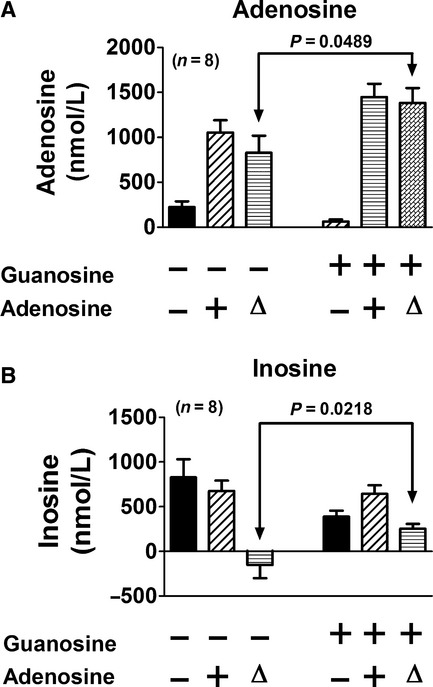
Bar graphs show (A) adenosine and (B) inosine levels in the renal venous perfusate before and during administration of adenosine (3 *μ*mol/L) either with or without coadministration of guanosine (30 *μ*mol/L). The change (Δ) in adenosine and inosine levels induced by the adenosine administration is also indicated. The *P*‐values are from paired Student's *t*‐tests comparing the change in adenosine or inosine in the absence and presence of guanosine. Values represent means and SEMs.

Having determined that energy depletion in the kidney does indeed increase guanosine, adenosine, and inosine, we next examined in a third experimental series whether guanosine can alter the clearance of adenosine by the isolated, perfused mouse kidney. After a 1‐h rest period, a 1‐min sample of venous perfusate was obtained, and then exogenous adenosine was added to the arterial perfusate (3 *μ*mol/L, final concentration in perfusate). After 5 min, another 1‐min sample of venous perfusate was collected during the adenosine administration. After a 30‐min washout period, this protocol was repeated, but this time in the presence of guanosine (30 *μ*mol/L, final concentration in perfusate). As shown in Figure 3, in the absence of guanosine, administration of adenosine into the renal artery increased renal venous levels of adenosine by 828 ± 190 nmol/L; however, in the presence of guanosine, administration of adenosine increased renal venous levels of adenosine by 1383 ± 165 nmol/L (*P* = 0.0489). In the absence of guanosine, administration of adenosine into the renal artery did not increase renal venous levels of inosine, which actually decreased by 152 ± 148 nmol/L. In contrast, in the presence of guanosine, adenosine increased inosine levels in the renal venous perfusate by 254 ± 53 nmol/L (*P* = 0.0218).

**Figure 4.**
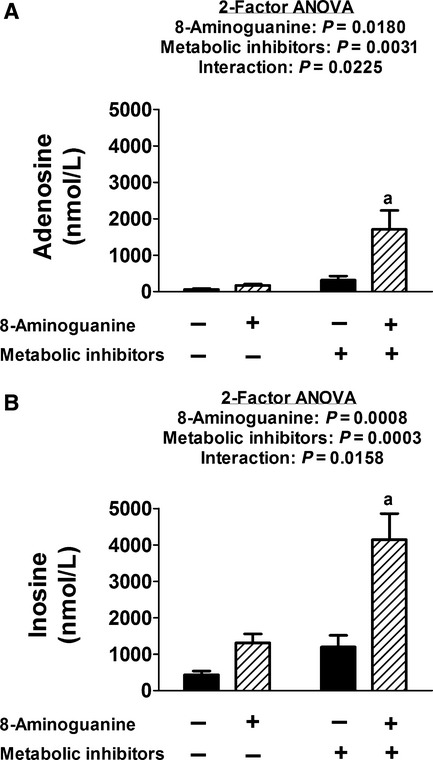
Bar graphs show the effects of metabolic inhibitors (iodoacetate and 2,4‐dinitrophenol, each at 50 *μ*mol/L for approximately 15 min) on renal venous concentrations of (A) adenosine and (B) inosine in isolated, perfused mouse kidneys in the absence (*n* = 8) and presence (*n* = 8) of 8‐aminoguanine (30 *μ*mol/L). Values represent means and SEMs. *P*‐values are from nested 2‐factor analysis of variance (2‐factor ANOVA) and “a” indicates *P* < 0.05 compared with all other groups.

In a fourth experimental series, we investigated whether modulation of endogenous guanosine levels would influence endogenous levels of adenosine and inosine. In these experiments, we isolated and perfused an additional 16 mouse kidneys. After a 1‐h rest period, eight kidneys were treated with 8‐aminoguanine (30 *μ*mol/L, final concentration in the arterial perfusate). 8‐Aminoguanine is a potent inhibitor of PNPase (Ki = 0.8 *μ*mol/L; Chern et al. 1993); and PNPase metabolizes guanosine to guanine and inosine to hypoxanthine, but is reported not to directly metabolize adenosine to adenine (Giblett 1985; Seegmiller 1985). After 15 min, a 1‐min sample of renal venous perfusate was collected and the kidneys were then treated with iodoacetate plus dinitrophenol to block energy production, and after 15 min, another 1‐min sample of renal venous perfusate was collected. As illustrated in Figure 4, metabolic poisons again increased renal venous levels of adenosine (*P* = 0.0031) and inosine (*P* = 0.0003); however, there was a significant interaction between 8‐aminoguanine and metabolic poisons on renal venous levels of both adenosine (*P* = 0.0225) and inosine (*P* = 0.0158) such that the effects of metabolic poisons on adenosine and inosine were markedly augmented by blocking PNPase.

## Discussion

By engaging A_1_, A_2A_, A_2B_, and A_3_ adenosine receptors (Jacobson 2009; Fredholm 2010; Trincavelli et al. 2010) adenosine influences most organ systems including the kidneys (Vallon et al. 2006), heart (Mustafa et al. 2009), liver (Peng et al. 2008), brain (Sebastiao and Ribeiro 2009), lungs (Mohsenin and Blackburn 2006; Vass and Horvath 2008), bladder (Yu et al. 2006, 2011), skeletal muscle (Hespel and Richter 1998; Marshall 2002), adipose tissue (Fredholm et al. 2011), autonomic nervous system (Westfall et al. 1990; Pelleg et al. 1997), and immune system (Bours et al. 2006; Kumar and Sharma 2009). Because adenosine receptors reside on the cell surface, the most relevant pool of adenosine for regulating organ function is that in the extracellular space. An important concept is that the levels of extracellular adenosine depend on a dynamic balance between those mechanisms that produce extracellular adenosine versus those processes that remove adenosine from the interstitial compartment (Grenz et al. 2012). Our previous studies in cell culture model systems show that extracellular guanosine is a potential endogenous regulator of adenosine disposition from the extracellular compartment (Jackson and Gillespie 2013; Jackson et al. 2013) and thereby may substantively contribute to higher levels of extracellular adenosine; but whether this “guanosine–adenosine mechanism” is operative in an intact organ system is an open question.

The results of the present study are consistent with the hypothesis that the guanosine–adenosine mechanism indeed does occur in an intact organ, namely the kidney. In this regard, four lines of evidence support the conclusion that the guanosine–adenosine mechanism exists in the intact kidney. First, we find that the time courses of appearance of guanosine, adenosine, and inosine (adenosine metabolite) in the renal vein (vascular compartment) following the administration of metabolic poisons are similar. Not only are the time courses for the appearance of renal venous guanosine and adenosine similar, so too are the magnitude of these changes (expressed as a percentage of basal levels). Notably, the percentage increase in renal venous inosine exceeds that for adenosine and guanosine, which is consistent with our previous finding that guanosine inhibits not only the disposition of adenosine but also the disposition of inosine (Jackson et al. 2013). Thus, guanosine potentially could elevate extracellular inosine via two mechanisms (1) augmentation of the levels of adenosine (which is inosine precursor); and (2) direct inhibition of inosine disposition. A second line of evidence supporting the hypothesis that the guanosine–adenosine mechanism exists in the intact kidney is the observation that in the tissue compartment (which would represent in part interstitial levels of purines) metabolic poisons increase the levels of guanosine, adenosine, and inosine; and once again inosine increases more than adenosine. These data indicate that stimuli that increase the extracellular levels of guanosine also increase extracellular levels of adenosine and inosine. Because extracellular guanosine is increased along with extracellular adenosine and inosine, guanosine has the opportunity to modulate adenosine and inosine levels. However, the association between guanosine and adenosine or inosine does not prove cause and effect since adenosine or inosine could be modulating guanosine levels or the stimulus that increases extracellular adenosine and inosine could also increase extracellular guanosine. Therefore, the present investigation uses additional approaches to confirm or refute our hypothesis.

One approach to test cause and effect is to examine whether exogenous guanosine alters the clearance (extraction) of exogenous adenosine by the intact kidney. Notably, our results show that coadministration of guanosine augments the effects of adenosine administration on renal venous levels of adenosine and its metabolite inosine. These experiments establish that guanosine in the vascular compartment can inhibit the renal extraction of adenosine and inosine, and this finding provides a third line of evidence supporting the guanosine–adenosine mechanism. However, a limitation of these experiments is that the interactions between exogenous guanosine and adenosine might not accurately mimic the interactions between endogenous guanosine and adenosine or inosine; so more evidence is required to confirm or refute the concept that the guanosine–adenosine mechanism exists in the intact kidney.

Accordingly, the present investigation describes yet a fourth line of evidence that the guanosine–adenosine mechanism exists in the intact kidney. Specifically, our results shows that the efficacy of metabolic poisons to increase renal venous adenosine is enhanced by the coadministration of 8‐aminoguanine, a drug that potently inhibits the metabolism of guanosine by PNPase. This finding supports the concept of the guanosine–adenosine mechanism because if this mechanism is operative, then inhibition of guanosine metabolism would increase endogenous extracellular levels of adenosine. 8‐Aminoguanine also increases the efficacy of metabolic poisons to stimulate renal venous inosine levels. Although this too is consistent with our previous report that extracellular guanosine inhibits the disposition of both extracellular adenosine and inosine (Jackson et al. 2013), because PNPase also directly metabolizes inosine to hypoxanthine, the increase in inosine after 8‐aminoguanine may be due, in part, to accumulation of inosine because of its impaired metabolism to hypoxanthine.

In our previous publications, and in this article as well, we use the word “disposition” to describe the guanosine–adenosine mechanism because this term covers a large mechanistic territory and therefore leaves open many nonexclusive possibilities for the underlying basis of the “guanosine–adenosine mechanism.” Conceivably, extracellular guanosine could modify the disposition of extracellular adenosine by inhibiting transporters that shuttle adenosine across cell membranes or enzymes that are involved in the metabolism of adenosine. In this regard, our recently published work shows that adenosine deaminase, adenosine kinase, S‐adenosylhomocysteine hydrolase, guanine deaminase, equilibrative nucleoside transporters (SLC29 family members, also called ENTs), and concentrative nucleoside transporters (SLC28 family members, also called CNTs) are not involved in the guanosine–adenosine mechanism (Jackson et al. 2013). As the classical pathways for adenosine disposition are not involved in the guanosine–adenosine mechanism, we considered and explored less traditional transport systems, and our subsequent published studies show that other candidate transporters, including SLC19A1, SLC19A2, SLC19A3, and SLC22A2, are not involved (Jackson and Gillespie 2013). Although negative, these findings are nonetheless important because they suggest that the underlying basis of the guanosine–adenosine mechanism is quite unique; and the present study is important because it shows that whatever this unique mechanism is, it is of considerable physiological significance since it occurs in an intact organ system. Demonstrating that the guanosine–adenosine interaction occurs in a more physiological setting than cell culture encourages us, and we hope others, to work toward elucidating the basis for the guanosine–adenosine interaction. Currently, we are investigating whether the guanosine–adenosine interaction involves membrane trafficking.

The current findings have physiological implications. Adenosine modulates many renal parameters including renal hemodynamics, renal excretory function and renin release (Vallon et al. 2006). As extracellular guanosine modulates extracellular levels of adenosine, renal guanosine production, release, and disposition may be involved in regulating normal renal function; and dysregulation of the guanosine–adenosine mechanism could participate in renal pathophysiology.

The current observations also have therapeutic implications. There is overwhelming evidence that adenosine and inosine protect against acute kidney injury (AKI; Maggio et al. 1980; Marberger et al. 1980; Fitzpatrick et al. 1981; Rothwell et al. 1981; Mathur and Ramsey 1983; Okusa et al. 1999, 2000, 2001; Lee and Emala 2000, 2002; Okusa 2002; Day et al. 2005; Lee et al. 2007; Grenz et al. 2008, 2012; Kim et al. 2009; Bauerle et al. 2011). Therefore, guanosine per se could be a useful drug for preventing or treating AKI by augmenting extracellular adenosine and inosine in the kidney; and since guanosine would increase adenosine and inosine in a site‐ and event‐specific manner, guanosine might be a useful therapeutic agent without the adverse systemic effects of adenosine or inosine. Indeed, Kelly et al. (2001) report, in a comprehensive study, that guanosine (30 mg/kg) completely inhibits the rise in serum creatinine and tubular epithelial apoptosis induced by bilateral renal ischemia/reperfusion in the mouse.

The present results also suggest that inhibitors of PNPase might be useful for the prevention or treatment of AKI. In this regard, the present study shows that inhibition of PNPase augments the ability of metabolic inhibitors to increase extracellular adenosine and inosine. This suggests that in injured tissues, such as renal tissue suffering from AKI, PNPase inhibitors would be protective, yet in uninjured, normal tissues PNPase inhibitors would have little effect.

In conclusion, the present experiments demonstrate that extracellular guanosine and extracellular adenosine (and its metabolite inosine) interact in the intact kidney such that guanosine increases the levels of extracellular adenosine and inosine. This interaction likely would lead to increased activation of renal adenosine receptors with physiological and pharmacological implications. The focus of future experiments will be to elucidate the mechanism of and physiological roles of the guanosine–adenosine interaction and to explore the pharmacological potential of guanosine and PNPase inhibitors for prevention and treatment of organ injury.

## Conflict of Interest

None declared.
